# Immunobiotic lactic acid bacteria beneficially regulate immune response triggered by poly(I:C) in porcine intestinal epithelial cells

**DOI:** 10.1186/1297-9716-42-111

**Published:** 2011-11-03

**Authors:** Shoichi Hosoya, Julio Villena, Tomoyuki Shimazu, Masanori Tohno, Hitomi Fujie, Eriko Chiba, Takeshi Shimosato, Hisashi Aso, Yoshihito Suda, Yasushi Kawai, Tadao Saito, Susana Alvarez, Shuji Ikegami, Hiroyuki Itoh, Haruki Kitazawa

**Affiliations:** 1 Food Immunology Group, Laboratory of Animal Products Chemistry, Graduate School of Agricultural Science, Tohoku University, Sendai 981-8555, Japan; 2 Laboratory of Animal Breading and Genetics, Graduate School of Agricultural Science, Tohoku University, Sendai 981-8555, Japan; 3 National Agriculture and Food Research Organization, National Institute of Livestock and Grassland Science, Nasushiobara 329-2793, Japan; 4 Department of Nutritional Physiology, Graduate School of Pharmaceutical Science, Josai University, Sakado 350-0295, Japan; 5 Fiber-Nanotech Young Researcher Empowerment Center, Shinshu University, Minamiminowa 399-4598, Japan; 6 Cell Biology Laboratory, Graduate School of Agricultural Science, Tohoku University, Sendai 981-8555, Japan; 7 Department of Food, Agriculture and Environmental Sciences, Miyagi University, Sendai 982-0215, Japan; 8 Laboratory of Clinical and Experimental Biochemistry, Reference Centre for Lactobacilli (CERELA-CONICET). Tucuman, Argentina; 9 Division of Research and Development, Meiji Corporation, Kanagawa 250-0862, Japan

## Abstract

This study analyzed the functional expression of TLR3 in various gastrointestinal tissues from adult swine and shows that TLR3 is expressed preferentially in intestinal epithelial cells (IEC), CD172a^+^CD11R1^high ^and CD4^+ ^cells from ileal Peyer's patches. We characterized the inflammatory immune response triggered by TLR3 activation in a clonal porcine intestinal epitheliocyte cell line (PIE cells) and in PIE-immune cell co-cultures, and demonstrated that these systems are valuable tools to study in vitro the immune response triggered by TLR3 on IEC and the interaction between IEC and immune cells. In addition, we selected an immunobiotic lactic acid bacteria strain, *Lactobacillus casei *MEP221106, able to beneficially regulate the anti-viral immune response triggered by poly(I:C) stimulation in PIE cells. Moreover, we deepened our understanding of the possible mechanisms of immunobiotic action by demonstrating that *L. casei *MEP221106 modulates the interaction between IEC and immune cells during the generation of a TLR3-mediated immune response.

## Introduction

The central challenge of the intestinal immune system is balancing defense with immunological tolerance, including responding to pathogens while coexisting with resident bacteria and food antigens. The proper balance of this cross-regulation in the gut involves the activity of immune cells and intestinal epithelial cells (IEC) that are able to distinguish the diverse elements of the intestinal environment by pattern recognition receptors (PRR) [1]. Toll-like receptors (TLR) are one of the most important PRR in innate immunity. TLR can recognize foreign bacterial cell-wall structures, genome DNA or viral DNA/RNA intermediates and play a critical role in pathogen recognition and host defense [2,3].

TLR3 is a receptor for double-strand RNA (dsRNA) and upon recognition of its ligand, TLR3 transmits signals that activate the transcription factors IRF-3, NF-kB, and AP-1, leading to the induction of type I interferons (IFN) (especially IFN-β) and cytokine/chemokine production [4]. The production of cytokines and chemokines by IEC and immune cells after TLR3 activation is one of the major innate immune responses against dsRNA viruses such as rotavirus. Rotavirus dsRNA triggers the production of IL-8, IP-10, IL-6, TNF-α and IL-15 in IEC via the TLR3-activated pathway inducing recruitment and activation of macrophages and NK cells and stimulating adaptive B- and T-cell immune responses [5,6]. Since TLR3 responds to a synthetic dsRNA, poly(I:C) as well as viral dsRNA resulting in the induction of IFN-α/β and IFN- inducible gene transcription, it is thought that TLR3 plays a key role in anti-viral immune responses [5].

The cascade of events triggered upon TLR3 engagement by viral and synthetic dsRNA has been extensively studied in different cell models and a number of events and key molecules involved, such as adaptor proteins and transcription factors have been described. The majority of these studies aimed at dissecting the mechanisms of TLR3 function have been mostly performed in mice and human cells [7]. Few studies have been performed in the swine. The full-length cDNA of porcine TLR3 was identified and characterized and it was found that TLR3 contains typical functional TLR domains and shares about 80% sequence identity to other mammalian orthologues [8]. Moreover, tissue expression profiles showed that porcine TLR3 is highly expressed in the kidney, duodenum, spleen and liver, and moderately expressed in bone marrow, lung and skin of adult pigs [8]. However, no further studies have been performed regarding the functional role of TLR3 in the immune responses against porcine viral diseases.

In recent years, there has been a growing interest in the swine immune system because of its possible use as a model for the human immune system and because of the economic importance of swine as livestock [2,3,9]. Therefore, investigating how TLR3 mediates microbial stimulation of the gut-associated lymphoid tissues (GALT) such as Peyer's patches (PP) in the swine would be important for understanding the activation and regulation of the intestinal immune system not only in pigs but also in humans. Moreover, understanding how TLR3 is activated and regulated in immune cells and IEC can help to choose effective therapies for the prevention or treatment of viral diseases in humans and pigs. For example, it could help to select certain strains of lactic acid bacteria (LAB) with immunomodulatory properties via intestinal immunity (immunobiotics) capable of protecting against such diseases by increasing viral defenses and preventing unproductive inflammatory response. In this sense, our laboratory recently developed in vitro systems using a clonal porcine intestinal epitheliocyte cell line (PIE cells) which could be a useful tool for the study of TLR3 activation on IEC and for the selection of LAB strains with specific immunomodulatory properties [10].

Considering this background, the aims of the current study were the following: i) to characterize the inflammatory immune response triggered by poly(I:C) stimulation in PIE cells and in PIE-immune cell co-cultures in order to evaluate if this in vitro model could be used as a valuable tool for the study of TLR3 activation on IEC; and ii) to select immunobiotic LAB strains able to beneficially modulate the anti-viral immune response triggered by TLR3 activation in IEC using PIE cell in vitro models.

## Materials and methods

### Quantitative expression analysis by real-time polymerase chain reactions (PCR) in swine tissues

Tissues (spleen, lung, duodenum, jejunum, ileum, ileal PP, MLN and colon) were obtained from adult (age 6 months) LWD swine (*n *= 9; genotype 1/4 Landrace, 1/4 Large White, 1/2 Duroc; Hiruzu Co., Ltd., Miyagi, Japan). The swine were clinically healthy and free of infectious diseases. The present study was conducted in accordance with the Guidelines for Animal Experimentation of Tohoku University, Japan.

Total RNA was isolated from the various adult swine tissues as described previously [11,12]. Briefly, cDNA were prepared by reverse transcription of 1 μg total RNA using Oligo d(T)18 primer (Invitrogen, Carlsbad, CA, USA). An equivalent volume of cDNA solution (5 μL) from each sample was used for quantification of porcine TLR3-specific cDNA. The real-time quantitative PCR reactions were performed with the 7300 Real-time PCR System (Applied Biosystems, Warrington, UK) using Platinum SYBR Green qPCR SuperMix UDG with ROX (Invitrogen, Tokyo, Japan) and the following primers: sense TAGAGACATGGATTGCTCCC, antisense AACTTCTGGAATGCAGGTCC. PCR was carried out with an initial denaturation for 10 min at 95 °C, followed by 45 cycles of 15 s at 95 °C, 10 s at 60 °C, and 5 s at 72 °C.

To quantify cytokine mRNA using real-time RT-PCR, cDNA standards were produced for TLR3 and β-actin as previously described [10]. Briefly, the cDNA were prepared by reverse transcription from 5 μg of total RNA using oligo (dT)18 primers (Invitrogen, Tokyo, Japan) and ThermoScript RNase H− reverse transcriptase (Invitrogen, Tokyo, Japan). The PCR products were inserted into the vector pGEM-T easy DNA (Promega, Madison, WI, USA). We confirmed the homology of each insert with dideoxy chain termination methods using a DNA sequencer (4000 L; Li-Cor, Lincoln, NE, USA) and the SequiTherm EXCELTM II DNA Sequencing Kit-LC (Epicentre Biotechnologies, USA). The cDNA standards were amplified by PCR from the vector pGEM-T easy DNA-TLR3 or -β-actin and standards were purified and quantified spectrophotometrically. The copy number for each mRNA was determined using the following formula: Copy number (copies) = 6.02 × 10^23 ^(copies/mol) × measurement (g)/MW (g/mol). MW = size in bp × 660 (g/mol/bp). Aliquots of standard cDNA containing 10^7^-10^2 ^fg/μL were created for β-actin and TLR3 for use as assay standards. TLR3 mRNA levels in different tissues were calibrated by the swine β-actin level, and normalized by common logarithmic transformation in comparison to the TLR3 mRNA level in the spleen (as 1.00). Values represent means and error bars indicate the standard deviations. The results are means of 3 measures repeated by 9 times of independent experiments using various tissues from at least 9 different adult swine. Amplification products of contaminants such as primer dimers were not detected by SYBR green chemistry using serial dilutions of cDNA.

### Analysis of TLR3 expression in porcine ileal PP cells

Expression levels of TLR3 protein in porcine ileal PP immunocompetent cells were determined by flow cytometry. Porcine PP immune single-cell suspensions were prepared from the ileum of adult swine as previously described [11,13-15]. Briefly, after cutting the specimens into small fragments, they were gently pressed through a nylon mesh and washed three times in complete RPMI 1640 medium (Sigma) supplemented with 10% FCS (Sigma). Residual erythrocytes were lysed by resuspension in hypotonic salt solution (0.2% NaCl), followed by hypertonic rescue in an equal volume of 1.5% NaCl. Finally, immune cells were fractionated with Lympholyte-Mammal (Cedarlane, Hornby, Ontario, Canada) density gradient centrifugation and the cells obtained were suspended in complete DMEM (Invitrogen, Tokyo, Japan) supplemented with 10% FCS (Sigma), 50 μg/mL penicillin/streptomycin and 50 μg/mL gentamycine (Nakalai tesque, Kyoto, Japan). This mononuclear cell suspension contains a mixed population of T (CD4^+ ^and CD8^+^), B (CD21^+^) and antigen presenting cells (CD4^- ^CD8^- ^MHCII^+^) [16].

For the detection of cell surface and intracellular TLR3 in the different populations of immune cells: leukocytes (CD45^+^), antigen-presenting cells (CD172a^+^CD11R1^-^, CD172a^-^CD11R1^low ^and CD172a^+^CD11R1^high^), B cells (CD21^+^) and T cells (CD4^+ ^and CD8^+^), the following primary antibodies were used: anti-mouse TLR3(unlabeled) rabbit-IgG (M-300:sc-28999, Santa Cruz Biotechnology, CA, USA), anti-porcine CD3(R-PE) mouse IgG1k (SouthernBiotech, 4510-09), anti-porcine CD4(unlabeled) mouse IgG2a (PT90A VMRD Inc., WA, USA), anti-porcine CD4(FITC) mouse IgG2b (4515-02, SouthernBiotech, Alabama, USA), anti-porcine CD8a(Biotin) mouse IgG2b (732844, Beckman Coulter, Tokyo, Japan) anti-porcine CD11R1(unlabeled) mouse IgG1 (MCA1220, AbD serotec, Kidlington, UK), anti-porcine CD172a(R-PE) SWC3 mouse IgG1 (4525-09, SouthernBiotech), anti-porcine CD172a(Biotin) SWC3 mouse IgG1 (4525-08, SouthernBiotech), and anti-porcine CD45RA(unlabeled) mouse IgG1 (PG96A, VMRD). In addition the following secondary antibodies were used: goat anti-rabbit IgG (H+L) Alexa Fluor488 (A-11008, Invitrogen, Tokyo, Japan), goat anti-mouse IgG2a-PE (sc-3765, Santa Cruz Biotechnology), goat anti-mouse IgG1-PerCP/Cy5.5 (sc-45103, Santa Cruz Biotechnology) and streptavidin(PE) (12-4317-87, eBioscience, CA, USA). Cells stained with irrelevant mouse IgG2b-FITC (11-4732, eBioscience), IgG1-PerCP/Cy5.5 (45-4714, eBioscience), IgG2b-PE (12-4732, eBioscience), IgG2a-PE (12-4724, eBioscience), IgG1-PE (12-4714, eBioscience) and rabbit IgG-Alexa Fluor488 isotype control (4340S, Cell Signaling Technology Japan KK, Tokyo, Japan) antibodies were included as isotype controls.

The cells were collected, washed twice with washing buffer (2% FCS, 0.01% NaN3/PBS) and the live cell counts were adjusted to 1 × 10^6 ^cells/tube. Immune cells were resuspended and labeled with primary and secondary antibodies for detection of CD3, CD4, CD8, CD21, CD45, CD172a and CD11R1. Finally, the cells were permeabilized with the BD cytofix-cytoperm kit (BD Biosciences, San Jose, CA, USA) according to the manufacturer's instructions and then labeled with primary anti-mouse TLR3(unlabeled) rabbit-IgG and secondary goat anti-rabbit IgG (H+L) Alexa Fluor488 antibodies for detection of intracellular TLR3. In addition, non-permeabilized immune cells were used to evaluate cell surface expression of TLR3.

Analysis of the stained cells was performed using FACS-Calibur™ (BD, Franklin Lakes, NJ, USA) equipped with Cell-Quest software. Data analysis was performed by using FlowJo software (Tree star, Ashland, Oregon, USA). For the analysis, fluorescence of permeabilized and non-permeabilized cells stained with anti-TLR3 antibody were compared simultaneously with isotype control and presented in the same histogram. In addition, intracellular and cell surface expression of TLR3 is presented as a log of MFI.

### Analysis of TLR3 expression in PIE cells

PIE cells, which are non-transformed intestinal cell lines originally derived from intestinal epithelia isolated from an unsuckled neonatal swine, were isolated and cloned previously [10]. When PIE cells are cultured, they assume a monolayer, cobblestone and epithelial-like morphology, with close contact between the cells. In addition, PIE cells are strongly positive for cytokeratin K8.13, a marker for porcine intestinal epithelial cells [10]. For the passage, PIE cells were treated with a sucrose/EDTA buffer for 4 min, detached using 0.04% trypsin in PBS, and then plated at 1.5 × 10^4 ^cells/cm^2 ^in culture flasks (Nalge Nunc International, Rochester, New York, USA) at 37 °C in an atmosphere of 5% CO_2_. PIE cells were cultured in 10% FCS DMEM and passaged every 3 or 4 days. In the present study, we used PIE generations from 20 to 30.

Immunohistochemical analysis of PIE cells was conducted according to our previous reports [10]. Briefly, PIE cells were grown on COL-coated cover glass (12 mm; Iwaki, Japan) in 24-well plates and cultured over-night. CellLight™ Early Endosomes-RFP BacMam 2.0 (Invitrogen) or Null control were added after culture according to the manufacturer's instruction, and cultured for 1 day to visualize endosomes. These cover glasses were then fixed in 4% paraformaldehyde solution in PBS for 20 min and permeabilized with 0.2% Triton X-100 in PBS for 5 min. PIE cells were then blocked with 2% normal goat-serum (Sigma) in PBS for 30 min. After removal of the blocking solution, PIE cells were incubated for 16 h at 4 °C with anti-mouse TLR3 (unlabeled) rabbit-IgG (Santa CruzBiotechnology, M-300:sc-28999) or rabbit-IgG isotype control (20304E, IMGENEX, CA, USA) in Can Get Signal^® ^immunostain solution (Toyobo, Tokyo, Japan), and then stained with secondary anti-rabbit IgG (H+L) Alexa Fluor488 (Invitrogen A-11008) antibody. After labeling, the cells were washed with PBS, rinsed in distilled water and mounted in Fluoroshield with DAPI (Gentaur, Kobe, Japan). The samples were analyzed using a confocal laser microscope (FluoView FV1000, OLYMPUS, Tokyo, Japan).

Flow cytometric analysis of PIE cells was conducted according to our previous reports [16]. PIE cells were collected and washed twice with washing buffer (2% FCS, 0.01% NaN3/PBS). The live cell counts were adjusted to 1 × 10^6 ^cells/tube and the cells were fixed and permeabilized with the BD cytofix-cytoperm kit (BD Biosciences, San Jose, CA, USA) according to the manufacturer's instructions. Then, fixed and permeabilized cells were resuspended and labeled with primary anti-mouse TLR3 (unlabeled) rabbit-IgG and secondary goat anti-rabbit IgG (H+L) Alexa Fluor488 antibodies for detection of intracellular TLR3. In addition, non-permeabilized PIE cells were used to evaluate cell surface expression of TLR3. Intracellular and cell surface expression of TLR3 is presented as log of MFI.

### Lactobacilli immunomodulatory activity in PIE cells

The following lactobacilli strains were used in this study: *Lactobacillus reuteri *MEP221101 and MEP221102, *Lactobacillus casei *MEP221103, TL2768, MEP221104, MEP221105, MEP221106, MEP221107, MEP221108, MEP221109, MEP221114 and MEP221115, *Lactobacillus rhamnosus *MEP221110, MEP221111, MEP221112 and GG, *Lactobacillus salivarius *MEP221113, *Lactobacillus jensenii *TL2937 and *Lactobacillus gasseri *MEP221117. The lactobacilli strains were grown in MRS medium (Difco, Detroit, USA) for 16 h at 37 °C and washed with PBS. Lactobacilli were re-suspended in DMEM, enumerated in a microscope using a Petroff-Hausser counting chamber, and stored at -80 °C until use.

PIE cells were plated at 1.5 × 10^4 ^cells/24 well plate on type I collagen-coated plates (Iwaki, Tokyo, Japan), and cultured for three days. After changing medium, lactobacilli (5 × 10^7 ^cells/mL) were added and 48 h later, each well was washed vigorously with medium at least 3 times to eliminate all the stimulants, and then stimulated with poly(I:C) for the time indicated.

We performed two-step real-time quantitative PCR to characterize the expression of mRNA in PIE cells [11,12]. Total RNA from each sample was isolated from the PIE cells using TRIzol reagent (Invitrogen). All cDNA were synthesized by the same method mentioned above. Real-time quantitative PCR was carried out with a 7300 Real-time PCR System (Applied Biosystems) using Platinum SYBR Green qPCR SuperMix UDG with ROX (Invitrogen). The primers used in this study were described previously [10]. The PCR cycling conditions were 2 min at 50 °C; followed by 2 min at 95 °C; then 40 cycles of 15 s at 95 °C, 30 s at 60 °C and 30 s at 72 °C. The reaction mixture contained 5 mL of the sample cDNA and 15 mL of the master mix including the sense and antisense primers. Cytokine mRNA levels were calibrated by the swine β-actin level and normalized by common logarithmic transformation. Values represent means and error bars indicate the standard deviations. The results are means of 3 measures repeated 4 times with independent experiments.

### Lactobacilli immunomodulatory activity in PIE-immune cell co-culture system

Porcine PP immune single-cell suspensions were prepared from the ileum of adult swine as described above [11,13,15]. In the Transwell culture system, PIE cells were seeded in the apical surface at a concentration of 1.5 × 10^5 ^cells/well in 12-well tissue culture plates (Transwell-Col. (PTFE), pore size 0.2 mm) while porcine PP immunocompetent cells were seeded in the basolateral compartment at a concentration of 2 × 10^7 ^cells/well. For the evaluation of the immunomodulatory activity of lactobacilli in the PIE-immune cell co-culture system, the apical surface containing PIE cells was stimulated with lactobacilli strains for 48 h and then washed twice with PBS. Finally, PIE cells were stimulated with poly(I:C) for 12 h. Studies of mRNA expression in PIE cells were performed as described above. In addition, levels of IL-1β, IL-2, IL-6, IL-10, IL-12p40, TNF-α, IFN-α, IFN-β, IFN-γ and TGF-β mRNA were studied in immune cells by using previously described primers [10].

### Statistical analysis

The statistical analysis for all data was performed using the GLM of SAS computer program (SAS, 1994). All of the means and standard deviations were calculated 3 times with repeated measurements and experiments. Normalized fold expression was calculated as the ratio of mRNA expression of cytokines to one of β-actin normalized by common logarithmic transformation of expression. To examine the significance of the fixed effect of repeated experiments, the REG procedure of SAS programs was used while considering the linear regression of expression on the repeated experiments. Comparisons among mean normalized fold expressions to evaluate relative mRNA expressions of cytokines in cells were carried out using one-way ANOVA for an effect of time or bacteria strains as a fixed effect, and then examined by the Fisher least significant difference (LSD) test. For these analyses, the *p *< 0.05 level was used to define significance.

## Results

### Expression of TLR3 in ileal PP from adult pigs

**Figure 1:**
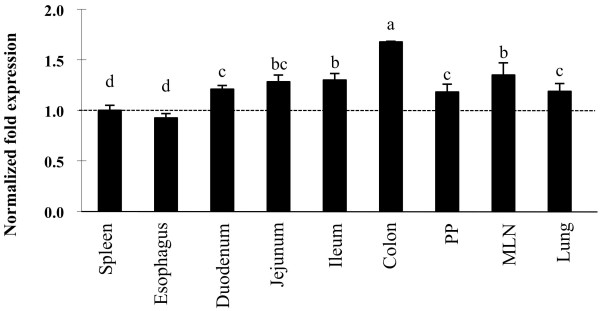
**Analysis of toll-like receptor 3 (TLR3) expression in tissues from adult swine**. TLR3 mRNA levels in different tissues were calibrated by the swine β-actin level, and normalized by common logarithmic transformation in comparison to the TLR3 mRNA level in the spleen (as 1.00). Values represent means and error bars indicate the standard deviations. The results are means of 3 measures repeated 9 times in independent experiments using various tissues from at least 9 different adult swine. The mean differences among different superscripts were significant at the 5% level.

We applied real-time quantitative PCR to analyze the expression of TLR3 mRNA in adult swine tissues (Figure 1). We previously used the strong expression in the spleen as a positive control to analyze the expression of TLR molecules in other tissues [11] therefore we performed a similar analysis for TLR3. The mRNA of TLR3 was expressed at detectable levels in all tissues of adult swine (Figure 1). TLR3 mRNA in adult pigs decreased in the following order: colon, MLN, ileum, jejunum, duodenum, lung, ileal PP, spleen and esophagus. Among the GALT of adult pigs, TLR3 mRNA was most strongly expressed in the MLN as in ileal PP.

**Figure 2:**
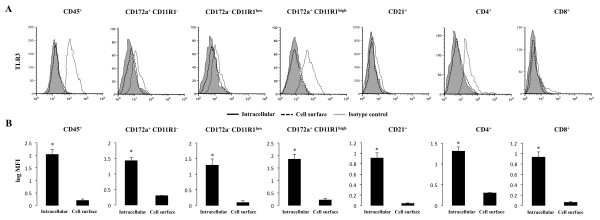
**Flow cytometric analysis of toll-like receptor 3 (TLR3) in immune cells from ileal Peyer's patches (PP)**. Mononuclear cells were isolated from adult swine PP and incubated with antibodies for CD45, CD172a, CD11R1, CD21, CD4 and CD8. (A) Histograms show flow cytometric analysis for TLR3 staining as follows: intracellular (open histogram), cell surface (broken lines) and isotype-matched controls (shaded histograms). (B) Values of log of mean fluorescence intensity (MFI) for intracellular and cell surface staining are shown. The results represent three independent experiments using ileal PP from at least three different swine.

PP are important for establishing effective immunological responses towards intestinal microbial antigens such as viruses. Since quantitative real-time PCR revealed that TLR3 mRNA is expressed in PP of adult swine, we next determined which cells contribute the response against TLR3 agonists in this tissue. TLR3 cell surface and intracellular expression was measured by flow cytometry in different populations of immune cells (Figure 2). We show that the leukocyte population (CD45^+ ^cells) expressed the TLR3 protein. The detailed study of different populations of lymphocytes B (CD21^+^) and T (CD4^+ ^and CD8^+^) cells demonstrated that TLR3 was expressed primarily by CD4^+ ^cells in adult pigs (Figure 2). In addition, we evaluated the expression of TLR3 in antigen presenting cells (APC). In swine, although monocytes/macrophages and DC in various tissues have been studied, no single specific markers are known that allow definitive identification. The most frequent marker on porcine macrophages and DC is CD172a. In addition, CD11R1 is considered to be a specifically and differentially expressed marker on porcine DC, but not in macrophages [9]. Moreover, DC emigrating from the intestine via lymphatic vessels in swine have been identified as CD172a^+^CD11R1^+ ^and CD172a^-^CD11R1^+ ^cells [17]. Thus, in the present study, we used CD172a and CD11R1 to define three populations of APC: CD172a^+^CD11R1^high^, CD172a^-^CD11R1^low ^and CD172a^+^CD11R1^- ^cells, according to our preliminary studies and previously published works [17-19]. For the study, high side-scatter cells were excluded by gating. In addition, expression of MHC-II was analyzed in CD172a^+^CD11R1^-^, CD172a^-^CD11R1^low ^and CD172a^+^CD11R1^high ^cells and we found that more that 80% of the cells in each population were MHC-II^+^. Within these three cell populations, we found that TLR3 is strongly expressed in CD172a^+^CD11R1^high ^cells (Figure 2). In addition, we did not find surface expression of TLR3 in any cell population (Figure 2).

### Functional expression of TLR3 in PIE cells

**Figure 3:**
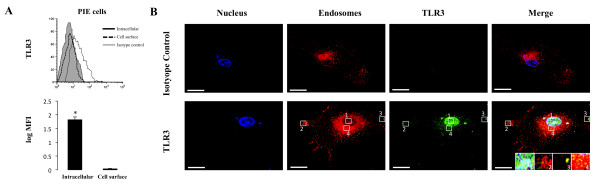
**Analysis of toll-like receptor 3 (TLR3) expression in porcine intestinal epithelial (PIE) cells**. (A) Flow cytometric analysis of TLR3 in PIE cells. Histograms show flow cytometric analysis for TLR3 staining as follows: intracellular (open histogram), cell surface (broken lines) and isotype-matched controls (shaded histograms). The analysis of intracellular and cell surface expression of TLR3 is presented as the log of mean fluorescence intensity (MFI). The results represent three independent experiments. (B) Confocal microscopic analysis of the subcellular localization of TLR3 in PIE cells. Fixed and permeabilized cells were stained with anti-mouse TLR3(unlabeled) rabbit-IgG and goat anti-rabbit IgG (H+L) Alexa Fluor488 and Early Endosomes-RFP BacMam. The merged color (yellow) is indicative of co-localization. Bar, 2 um. PIE cells stained with rabbit IgG-Alexa Fluor488 Isotype were used as controls.

**Figure 4:**
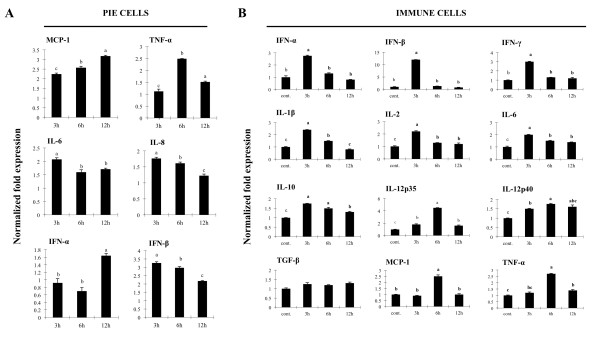
**Expression of cytokines in porcine intestinal epithelial (PIE) cells and immune cells**. (A) Monocultures of PIE cells were stimulated with poly(I:C) and the expression of IFN-α, IFN-β, IL-6, IL-8, MCP-1 and TNF-α was studied at the indicated times post stimulation. (B) Co-cultures of PIE cells and ileal PP immune cells were stimulated with poly(I:C) and the expression of IFN-α, IFN-β, IFN**-γ**, IL-12p40, TNF-α, IL-1β, IL-2, IL-6, IL-10 and TGF-β was studied in immune cells at the times indicated post stimulation. Cytokine mRNA levels were calibrated by the swine β-actin level and normalized by common logarithmic transformation. Values represent means and error bars indicate the standard deviations. The results are means of 3 measures repeated 4 times with independent experiments. The mean differences among different superscripts were significant at the 5% level.

In order to study the mechanisms by which IEC induce an immune response to microbial-associated molecular patterns, we previously established a clonal porcine intestinal epitheliocyte cell line (PIE cells). Studies of TLR1-9 and MD-2 mRNA expressions on PIE cells demonstrated that TLR4 was most strongly expressed, followed by TLR3 [10]. In order to evaluate the expression of TLR3 protein in PIE cells, we performed flow cytometry analysis using the anti-mouse TLR3 antibody. We observed that TLR3 was detected only by intracellular staining (Figure 3a). To confirm the intracellular expression of TLR3 in PIE cells, immunohistochemical staining was performed and analyzed by confocal microscopy. As shown in Figure 3b, TLR3 was strongly expressed in the cytoplasm of PIE cells and it was detected together with some early endosomes while it was not detected in the isotype control. Delineating cytokine and chemokine responses to TLR3 stimulation would be important to understand TLR3 mediated immune response and pathogenicity in IEC. Thus, we next evaluated the response of PIE cells to the stimulation with poly(I:C). Challenge of PIE cells with the TLR3 agonist significantly increased the expression of the pro-inflammatory cytokines and chemokines (Figure 4a). Levels of IL-6 and IL-8 mRNA increased after stimulation with poly(I:C) reaching a maximum value at hour 3 (2 and 1.7 normalized folds respectively), while TNF-α exhibited a peak at 6 h (2.4 folds). On the contrary, MCP-1 reached its highest level of expression at hour 12 (3.1 folds). In addition, there was an increase in the levels of IFN-α and IFN-β after the challenge of PIE cells with poly(I:C) (Figure 4a). IFN-β mRNA levels were strongly up-regulated (3.2 folds) from hour 3 post-stimulation. Although a progressive decrease in the levels of IFN-β was observed after hour 3, this cytokine was significantly increased at hours 6 (2.9 folds), 12 (2.1 folds) (Figure 4a) and 48 (1.7 folds) (data not shown). In addition, IFN-α was up-regulated after challenge with poly(I:C) reaching the highest levels of expression at hour 12 (1.6 folds). We also evaluated the expression of TLR3 in PIE cells challenged with poly(I:C) and found that TLR3 was up-regulated 1.1, 1.6 and 1.9 folds at hour 3, 6 and 12 respectively (data not shown).

The activation of innate immune signaling in the intestinal epithelium modulates adaptive immunity through cross-talk with regulatory immune and effector cells in the lamina propria. Therefore, we next evaluated the response of PIE cells and PP immune cell co-cultures to the challenge with poly(I:C). The response of the PIE cells in co-culture was similar (data not shown) to that found in the monocultures of PIE cells stimulated with poly(I:C) (Figure 4a). On the contrary, the stimulation of co-cultures with poly(I:C) increased the expression of pro-inflammatory cytokines TNF-α (2.7 folds), IL-1β (2.4 folds), MCP-1(2.5 folds) and IL-6 (2 folds) in immune cells (Figure 4b). An early increase in the expression of IFN-α, IFN-β, IFN-γ, IL-2 and IL-12p40 (2.7, 12, 3.1, 2.2 and 1.5 folds at hour 3 respectively) was also observed in immune cells (Figure 4b). When the expression of the immunoregulatory cytokines IL-10 and TGF-β were studied, we observed an increase in levels of IL-10 from 3 h post-stimulation (1.7 folds), whereas TGF-β expression was unchanged throughout the whole time studied (Figure 4b).

### Selection of immunobiotic LAB in PIE cells

**Figure 5:**
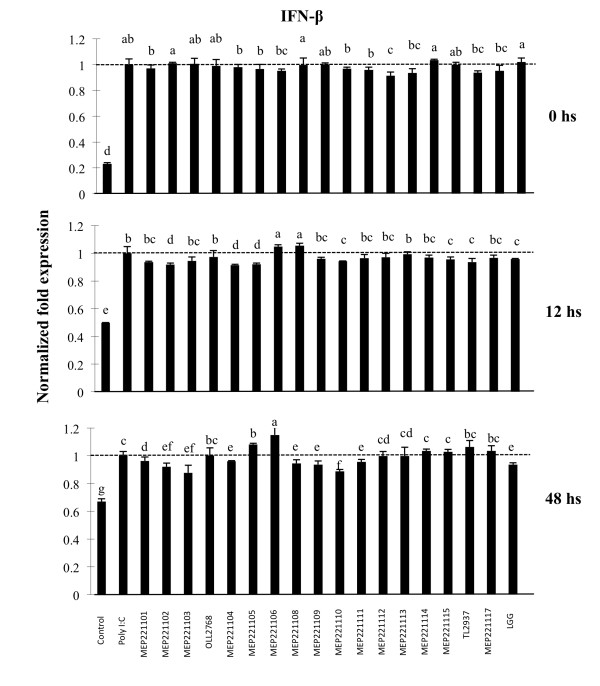
**Selection of immunomodulatory lactobacilli**. Porcine intestinal epithelial (PIE) cells were pre-treated with lactobacilli for 48 h, stimulated with poly(I:C) and then the expression of IFN-β was studied at different times post-stimulation. IFN-β mRNA levels was calibrated by the swine β-actin level, and normalized by common logarithmic transformation in comparison to the IFN-β mRNA level in the poly(I:C) (as 1.00). Values represent means and error bars indicate the standard deviations. The results are means of 3 measures repeated 3 times with independent experiments. The mean differences among different superscripts were significant at the 5% level.

We next employed the PIE cell in vitro system to select strains of LAB with immunoregulatory capabilities. IFN-β is an important cytokine for protection against viral infections, thus we selected strains able to improve the production of IFN-β in PIE cells stimulated with the TLR3 agonist. PIE cells were stimulated with different strains of LAB for 6, 12 or 48 h and then challenged with poly(I:C) (Figure 5). No significant differences in levels of IFN-β were found in PIE cells treated with the different lactobacilli at hour 0. The study of IFN-β mRNA expression levels revealed that only MEP221108 (1.05 fold) and MEP221106 (1.04 fold) strains were able to increase levels of IFN-β when compared to the control at hour 12. In addition, levels of IFN-β were increased 1.15 fold in PIE cells treated previously with MEP221106 strain at hour 48 (Figure 5). Considering that *L. casei *MEP221106 was the only strain capable of increasing IFN-β levels with 12 and 48 h of stimulation, this strain was selected for further studies. We also selected *L. casei *MEP221103 as a negative control strain.

**Figure 6:**
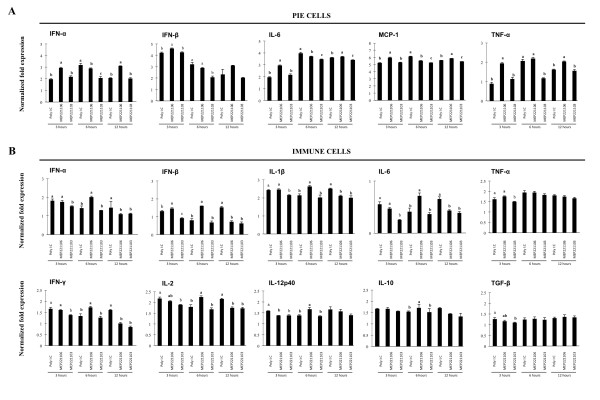
**Effect of lactobacilli in porcine intestinal epithelial (PIE) and immune cell co-cultures**. PIE cells were co-cultured with ileal PP immunocompetent cells, pre-treated with *L. casei *MEP221106 or *L. casei *MEP221103 for 48 h and then stimulated with poly(I:C). Cytokines were studied at the times indicated post-stimulation. (A) Expression of IFN-α, IFN-β, IL-6, MCP-1 and TNF-α in PIE cells. (B) Expression of IFN-α, IFN-β, IFN-γ, IL-12p40, TNF-α, IL-1β, IL-2, IL-6, IL-10 and TGF-β in immune cells. Cytokine mRNA levels were calibrated by the swine β-actin level and normalized by common logarithmic transformation. Values represent means and error bars indicate the standard deviations. The results represent three independent experiments. Values with different superscript letters in the same time post-poly(I:C) stimulation are significantly different at the 5% level.

Then we used the in vitro PP model culture system to evaluate the effect of *L. casei *MEP221106 more precisely. Co-cultures of PIE and immune cells were treated with *L. casei *MEP221106 or MEP221103 and then stimulated with poly(I:C). Expression of type I IFN and pro- and anti-inflammatory cytokines were measured at different times post-stimulation (Figure 6). *L. casei *MEP221106 significantly increased levels of IFN-α (2.9, 3.1 and 3.1 folds at hours 3, 6 and 12 respectively) and IFN-β (4.6 and 3.1 folds at hour 3 and 12 respectively) produced by PIE cells in response to the stimulus with poly(I:C). On the contrary, the negative *L. casei *MEP221103 strain did not improve the levels of these cytokines when compared with the control at hour 3 and significantly reduced the levels of IFN-α and IFN-β at hour 6 (Figure 6a). In addition, we observed that the stimulation of the PIE cell with poly(I:C) increased levels of IL-6, MCP-1 and TNF-α which reached a peak at hour 6. The treatment with *L. casei *MEP221106 induced an earlier and stronger increase in the levels of TNF-α, MCP-1 and IL-6 (1.9, 5.9 and 2.9 folds at hour 3 respectively) (Figure 6a). IL-6 and MCP-1 in *L. casei *MEP221106 treated cells were decreased when compared to controls at hour 6, while the three pro-inflammatory cytokines were improved again at hour 12 (Figure 6a). The negative control strain was able to induce significant reductions of TNF-α, MCP-1 and IL-6 at hour 6 (Figure 6a). When studying the expression of cytokines in immune cells we observed that the most significant changes induced by *L. casei *MEP221106 were produced at hour 6 (Figure 6b). The levels of IFN-α (2 folds), IFN-β (1.6 folds), IFN-γ (1.7 folds), IL-2 (2.2 folds), IL-12p40 (1.6 folds) and IL-1β (2.6 folds) in immune cells were significantly higher in cells stimulated with *L. casei *MEP221106 (Figure 6b). In addition, higher levels of IFN-β were observed in immune cells treated with *L. casei *MEP221106 at hour 3 (1.4 folds) (Figure 6b). On the contrary, *L. casei *MEP221106 induced down-regulation of IFN-α, IL-1β, IL-6, IFN-γ and IL-2 at hour 12 (Figure 6b). The treatment of co-cultures with *L. casei *MEP221103 did not improve the levels of the cytokines studied. This treatment induced a down-regulation of IFN-β at hour 3 and IFN-α, IL-1β, IL-6, IFN-γ and IL-2 at hours 3 and 12. When we evaluated the changes in the immunoregulatory cytokines, we observed that only the *L. casei *MEP221106 strain induced increases in expression levels of IL-10 (1.7 folds), while no changes were observed in the levels of TGF-β with either treatment (Figure 6b).

## Discussion

The entire coding sequences of porcine TLR1-TLR10 genes appeared in public nucleotide databases. Moreover, the genes encoding molecules utilized in transduction of the TLR signal in pigs, such as MyD88, have been cloned and characterized [20]. In this sense, we demonstrated that within the intestinal tissues and GALT of swine, TLR2 and TLR9 are strongly expressed in the ileal PP and MLN [11,13]. However, the expression of TLR3 in swine tissues has not been studied thoroughly. In the present work, we measured TLR3 mRNA in various tissues of adult swine and found that this receptor is strongly expressed in intestinal tissues and GALT. TLR3 is located in a variety of cell types of both mouse and human origin. However, both species- and cell type-specific differences in the expression and transcriptional regulation, as well as in cellular localization have been reported [7]. Thus, we next analyzed the functional expression of TLR3 protein in immune cells from ileal PP. We demonstrated that TLR3 is strongly expressed in CD172a^+^CD11R1^high ^cells, whereas expression levels of this receptor were significantly lower in the other populations of APC. Our study shows that in swine, TLR3 is strongly expressed in a subpopulation of APC from PP. This supports observations in humans and mice, since the analysis of TLR3 mRNA demonstrated that this transcript is selectively expressed in DC of the myeloid lineage but not in plasmacytoid DC [6]. Moreover, TLR3 is selectively expressed in human monocyte-derived DC and BDCA1^+ ^blood myeloid DC while in murine DC, TLR3 is present in the CD8a^+^CD4^- ^subset and bone marrow-derived DC [7]. The lymphoid lineage cells have also been reported to express TLR3. In humans, TLR3 mRNA was detected in peripheral blood T cells [21] and in mice, resting γδ T lymphocytes express TLR3 intracellularly [22]. Moreover, sorted naive CD45RB^high^CD4^+ ^T cells from mice expressed TLR3 even though this receptor is not present in highly purified CD8^+ ^lymphocytes [23]. In our study, we found a strong expression of TLR3 in porcine CD4^+ ^cells while a weak expression was found in CD8^+ ^cells from swine PP.

Epithelial TLR expression is also thought to be important for host defense against pathogens. IEC rather than other cell types express TLR3 in many organs including the airways and gastrointestinal tracts [24]. Human IEC expressed low levels of TLR2 and TLR4, while TLR3 seems to be abundantly expressed in normal human small intestine and colon [24]. By immunohistochemical and flow cytometric analysis, we observed an abundant intracellular expression of TLR3 in PIE cells. This was in line with findings of Liu et al. [25] that demonstrated that the non-transformed porcine jejunum epithelial cell line (IPEC-J2) constitutively expresses TLR3.

Several cytokines such as IL-1β, IL-6, IL-8, TNF-α and MCP-1 that are constitutively expressed by the IEC can be markedly up-regulated in response to microbial infection [1,10], therefore we next evaluated the response of PIE cells to poly(I:C) challenge. MCP-1, IL-8, TNF-α, IL-6 and both IFN-α and IFN-β were up-regulated in PIE cells after poly(I:C) stimulation. Moreover, considering that the interaction between IEC and immune cells is of fundamental importance for the type of immune response resulting from contact with an intestinal antigen, we also analyzed the expression of cytokines in immune cells by using co-cultures of PIE cells and porcine PP immune cells. After stimulation of co-cultures with poly(I:C), we observed an up-regulation of all the cytokines studied in immune cells with the exception of TGF-β. Then, the activation of TLR3 in PIE cells would induce the expression and release of cytokines, which exert their action on the underlying immune cells, inducing the activation of APC and effector lymphocytes. These results indicate that PIE cells could be a good tool to study in vitro immune response triggered by TLR3 on IEC and the interaction between IEC and immune cells.

The present study also showed that our in vitro systems could be used for the selection of immunobiotic LAB strains with anti-viral immune enhancing activities. The beneficial effects of immunobiotics on viral-associated diarrheas have been well established in humans [26]. However, few studies have evaluated their antiviral and anti-inflammatory effects in animals. Zhang et al. studied the effect of probiotics on the response of gnotobiotic pigs to the challenge with human rotavirus [18]. Although no differences were found between treated and control groups when virus replication was studied, the work suggested that LAB administration down-regulates the recruitment of viral-activated monocytes/macrophages into the intestinal tract thereby limiting the inflammation induced by the viral infection [18]. Later, it was shown that probiotic LAB had a significant influence on IFN-α, TGF-β, IL-4 and IFN-γ serum responses induced by rotavirus infection in gnotobiotic pigs [19]. In addition, recent studies by Maragkoudakis et al., [27] demonstrated that the known probiotics *L. casei *Shirota and *L. rhamnosus *GG were able to protect porcine and goat epithelial cells against rotavirus and transmissible gastroenteritis virus challenges, however, the immunological mechanisms involved in the protective effect were not investigated. In this study we aimed at selecting LAB strains able to improve anti-viral response. Among the lactobacilli strains evaluated, *L. casei *MEP221106 was the strain with the highest capacity to improve IFN-β production in poly(I:C)-challenged PIE cells. Moreover, in vitro co-culture experiments showed that *L. casei *MEP221106 is able to improve not only the production of IFN-β but also the levels of IFN-α, TNF-α, MPC-1 and IL-6 in PIE cells. In addition, immune cells in the co-cultures stimulated with *L. casei *MEP221106 showed and improved production of inflammatory and anti-viral cytokines when compared with control cells.

The modulation of cytokines induced by *L. casei *MEP221106 could have an important in vivo impact on viral intestinal infections such as those caused by rotavirus. Numerous studies have noted that rotaviruses are able to induce expression of type I INF in IEC and NK cells and that these responses contribute to innate immune-mediated clearance of viruses [6,28]. Type I and II IFN are able to limit rotavirus infection in vitro and in vivo studies have demonstrated that IFN-α administration is able to reduce rotavirus-associated diarrhea in cattle and pigs [29,30]. In addition, it was shown in rotavirus-infected mice that DC from PP had an increased expression of IL-12/23p40, INF-β and TNF-α, as well as the regulatory cytokine IL-10, suggesting that DC from PP play a critical role in controlling the infection and, at the same time, avoiding an excessive inflammatory immune response [24]. Studies in mice also showed that CD8^+ ^T cells are responsible for shortening the course of rotavirus primary infection [31] and that CD4^+ ^T cells are involved in supplying help to CD8^+ ^T cells, but also appear to mediate active protection, via an IFN-γ-dependent pathway [32]. Therefore, the results presented in this study suggest that *L. casei *MEP221106 would be capable of increasing anti-viral defenses in IEC (IFN-β and IFN-α) as well as the response of APC (IL-1β, IL-6, IL-12 and IL-10) and CD4^+ ^effector cells (IFN-γ).

In addition, we showed that immune cells in co-cultures pretreated with *L. casei *MEP221106 induced higher levels of IL-10 when compared with control and *L. casei *MEP221103 treated cells. IL-10 is a potent immunoregulatory cytokine that might be beneficial in the course of infection by attenuating the excessive host inflammatory response induced by up-regulated pro-inflammatory cytokines. It was reported that both purified viral dsRNA and poly(I:C) are able to induce severe mucosal damage via TLR3-dependent manner [33]. Moreover, it was shown that TLR3 is able to mediate harmful inflammatory responses in the intestine, thus contributing to the pathogenesis of viral infections [34]. While TLR3 recognition of dsRNA is required for clearance of viruses, it is believed that the degree and the duration of the pro-inflammatory cytokine secretion can become harmful to the host. Therefore, the improved production of IL-10 induced by *L. casei *MEP221106 would allow an efficient regulation of the inflammatory response and avoid tissue injury. As observed with other immunobiotic strains [35,36], *L. casei *MEP221106 up-regulated the expression of both pro- and anti-inflammatory cytokines. The simultaneous up-regulation of both types of cytokines could be explained by the response of a distinct population of immune cells. It was shown that DC from mice PP can increase the mRNA expression of IFN-β, IL-12/23p40, TNF-α and IL-10 genes after the challenge with rotavirus [37]. The dome- resident CD11c^+^CD11b^+^CD8^- ^DCs produce high levels of IL-10 upon stimulation while the interfollicular CD11c^+^CD11b^-^CD8^+ ^DC and the dome CD11c^+^CD11b^-^CD8^- ^DC produce mainly type I IFN and IL-12 [38]. Further studies are required to determine the role of porcine PP immune cell subsets in the response to poly(I:C) stimulation in order to obtain a more complete panorama of the mucosal immune response in swine and to understand the possible mechanisms of action of immunobiotic LAB. Moreover, the immunomodulatory activity of *L. casei *MEP221106 should be proven in appropriate experiments in vivo in order to conclusively asseverate that this bacterium is able to enhance antiviral immunity and protect against the inflammatory damage at the same time. These studies are currently under investigation in our laboratory.

The rapid expansion of mechanisms of communication between IEC, enteric commensals, probiotics and pathogens and the immune system has opened up exciting new avenues of research in humans and animals. This communication between host cells and microorganisms is mediated by PRR such as TLR. In this study we show that TLR3 is expressed in the gastrointestinal tract of pigs. The strong expression of this receptor in PP would indicate that it has an active participation in intestinal immune responses. We characterized the immune response triggered by TLR3 activation in PIE cells and in PIE-immune cell co-cultures, and demonstrated that these systems are valuable tools to study in vitro immune response triggered by TLR3 on IEC and the interaction between IEC and immune cells. In addition, we selected an immunobiotic LAB strain able to beneficially regulate the anti-viral immune response triggered by TLR3 activation in PIE cells and we deepened in the understanding of the mechanisms of immunobiotic action by studying the interaction between IEC and immune cells during the generation of a TLR3-mediated immune response. Our co-culture model could provide additional information on immune effects of different immunobiotic candidates such as *L. casei *MEP221106 since the modulatory role of IEC is included. Therefore, it may provide a useful tool to predict the in vivo effectiveness of immunobiotic strains.

## Competing interests

The authors declare that they have no competing interests.

## Authors' contributions

SH and JV designed and did most of the experiments and wrote the manuscript. TS and MT designed experiments and supervised the study. HF and EC carried out the anti-inflammatory assay. TS and HA supervised the study. YS performed the statistical analysis. YK, TS and SA supervised the study and discussed results. SI and HI prepared the experimental bacterial samples. HK designed experiments, wrote the manuscript and supervised the study. All authors read and approved the final manuscript.
